# The soluble (Pro) renin receptor does not influence lithium‐induced diabetes insipidus but does provoke beiging of white adipose tissue in mice

**DOI:** 10.14814/phy2.13410

**Published:** 2017-11-15

**Authors:** Kevin T. Yang, Fei Wang, Xiaohan Lu, Kexin Peng, Tianxin Yang, J. David Symons

**Affiliations:** ^1^ Department of Internal Medicine University of Utah Salt Lake City Utah; ^2^ College of Health University of Utah Salt Lake City Utah; ^3^ Molecular Medicine Program University of Utah Salt Lake City Utah; ^4^ Research Service Veterans Affairs Medical Center Salt Lake City Utah; ^5^ Institute of Hypertension Sun Yat‐sen University Zhongshan School of Medicine Guangzhou China

**Keywords:** Acquired nephrogenic diabetes insipidus, AQP2, beiging, collecting duct, lithium, sPRR

## Abstract

Earlier we reported that the recombinant soluble (pro) renin receptor sPRR‐His upregulates renal aquoporin‐2 (AQP2) expression, and attenuates polyuria associated with nephrogenic diabetes insipidus (NDI) induced by vasopressin type 2 receptor (V2R) antagonism. Patients that receive lithium therapy develop polyuria associated NDI that might be secondary to downregulation of renal AQP2. We hypothesized that sPRR‐His attenuates indices of NDI associated with lithium treatment. Eight‐week‐old male C57/BL6 mice consumed chow supplemented with LiCl (40 mmol/kg diets) for 14 days. For the last 7 days mice received either sPRR‐His [30 *μ*g/(kg day), i.v.; sPRR] or vehicle (Veh) via minipump. Control (Con) mice consumed standard chow for 14 days. Compared to Con mice, 14‐d LiCl treatment elevated water intake and urine volume, and decreased urine osmolality, regardless of sPRR‐His or Veh administration. These data indicate that sPRR‐His treatment does not attenuate indices of NDI evoked by lithium. Unexpectedly, epididymal fat mass was lower, adipocyte UCP1 mRNA and protein expression were higher, and multilocular lipid morphology was enhanced, in LiCl‐fed mice treated with sPRR‐His versus vehicle. The beiging of white adipose tissue is a novel metabolic benefit of manipulating the sPRR in the context of lithium‐induced NDI.

## Introduction

Lithium is a metallic monovalent cation that has been used for >150 years as a simple and effective treatment for psychiatric illnesses and Alzheimer's disease (Khanna 2006). Other human maladies wherein lithium has demonstrated therapeutic benefit include neurodegenerative diseases (Forlenza et al. 2014; Khasraw et al. 2012), migraine and cluster headache (Peatfield 1981), leukopenia (Focosi et al. 2009), and arthritis (El‐Mallakh and Jefferson 1999). Currently, ~30% of bipolar patients in the United States receives lithium therapy (Jamison 2000; Kishore and Ecelbarger 2013).

Despite its proven efficacy and affordability, lithium use has diminished over the past several decades due to adverse off‐target side effects that include nephrotoxicity (Nielsen et al. 2008b; Walker 1993). In this regard, ~70% of patients that receive lithium therapy develop nephrogenic diabetes insipidus (NDI) characterized by polyuria, polydipsia, and reduced urine osmolality due to impaired urine concentrating capability (Bedford et al. 2003; Timmer and Sands 1999). Although diverse mechanisms might be involved, the downregulation of renal AQP2 is considered to play a major role in the pathogenesis of NDI (Kwon et al. 2000; Marples et al. 1995). At present no effective therapy exists to manage lithium‐induced NDI.

Knowledge is evolving concerning the (pro) renin receptor (PRR), newly appreciated component of the renin‐angiotensin system (Nguyen et al. 2002) that serves as a specific receptor for prorenin and renin (Nguyen et al. 2002). A 28 kDa soluble form of PRR (sPRR), corresponding to the extracellular domain, is generated by protease mediated cleavage of the full‐length PRR, and circulates in the plasma (Cousin et al. 2009). A large number of clinical studies suggest that the circulating sPRR might be an important disease biomarker (Watanabe et al. 2012, 2013)^,^(Fukushima et al. 2013)^,^ (Bonakdaran et al. 2016)^,^(Nishijima et al. 2016)^,^ (Kreienbring et al. 2016), and we recently provided the first evidence for its biological relevance (Khanna 2006). In this regard, we generated a histidine‐tagged recombinant sPRR (i.e., sPRR‐His) and observed that sPRR‐His increased renal AQP2 expression and thereby improved the defective urine concentrating capability of mice with NDI secondary to V2R antagonism (Lu et al. 2016). This finding prompted us to test the efficacy of sPRR‐His in treating the defective urine concentrating capability of mice with NDI secondary to lithium treatment.

## Methods

### Animals and treatment groups

The protocol for this study was approved by the Institutional Animal Care and Use Committee at the University of Utah. Eight‐week‐old male C57BL/6J mice were purchased from The Jackson Laboratory (Bar Harbor, ME) and were housed in an environmentally controlled facility having a 12 h/12 h light/dark cycle. Animals had free access to tap water and standard rat chow. Mice were randomized into three groups that received 14 day treatment of either: (1) standard laboratory chow + no infusion; (2) LiCl (40 mmol/kg diets) + vehicle (saline) infusion (i.v.) for the last 7 days; or (3) LiCl [as in (2)] + with sPRR‐His infusion (30 *μ*g/(kg day), i.v.) for the last 7 days. sPRR‐His was administered via osmotic mini‐pump and this dose and duration is sufficient to upregulate renal AQP2 expression and prevent indices of NDI evoked by V2R antagonism in mice (Lu et al. 2016). Mice were relocated to metabolic cages for days 7–14 of each protocol. 24‐h water intake, urine volume, and urine osmolality were recorded on day 14.

On Day 15 mice were anesthetized with 2% isoflurane. Blood was withdrawn from the vena cava and both kidneys were excised. One kidney was snap frozen in liquid nitrogen after being sectioned into cortex and inner medulla, while the other was fixed in 4% PFA for 48 h and paraffin embedded. One epididymal fat pad was excised and weighed while 50% of the second was fixed and paraffin embedded and 50% was snap frozen in liquid nitrogen and used for immnoblotting and qRT‐PCR.

### Immunoblotting

White adipose tissue (WAT) was homogenized in cell lysis buffer and extracted as previously described (Park et al. 2015). Renal tissues were lysed and subsequently sonicated in PBS that contained 1% Triton x‐100, 250 *μ*mol/L phenylmethanesulfonyl fluoride (PMSF), 2 mmol/L EDTA, and 5 mmol/L dithiothrietol (DTT) (pH 7.5). Protein concentrations were determined by the use of Coomassie reagent. Forty microgram of protein for each sample was denatured in boiling water for 10 min, then separated by SDS‐PAGE, and transferred onto nitrocellulose membranes. The blots were blocked for 1 h with 5% nonfat dry milk in Tris‐buffered saline (TBS), followed by incubation overnight with primary antibody. After washing with TBS, blots were incubated with goat anti‐rabbit/mouse horseradish peroxidase (HRP)‐conjugated secondary antibody and visualized using Enhanced Chemiluminescence (ECL). Densitometry was quantified using Image‐Pro Plus (Media Cybernetics). Primary antibodies included: goat anti‐AQP2 antibody (Cat#sc‐9882,Santa Cruz Inc, Dallas, TX), and rabbit anti‐UCP1 (Cat# ab10983, Abcam Inc, Cambridge, MA). Renal AQP2 was normalized using *β*‐actin as a protein loading control. For immunoblotting of UCP1 in apididymal fat, Ponceau red staining was performed to validate equal loading.

### Quantitative RT‐PCR

Total RNA was isolated from renal tissues and WAT and reverse transcribed to cDNA. Oligonucleotides were designed using Primer3 software (bioinfo.ut.ee/primer3‐0.4.0/). Primers of AQP2 were 5′‐gctgtcaatgctctccacaa‐3′ (sense) and 5′‐ggagcaaccggtgaaataga‐3′ (antisense); primers of UCP1 were 5′‐cacggggacctacaatgctt‐3′ (sense) and 5′‐taggggtcgtccctttccaa‐3′ (antisense); primers for GAPDH were 5′‐gtcttcactaccatggagaagg‐3′ (sense) and 5′‐tcatggatgaccttggccag‐3′ (antisense).

### Hct measurement

Hct was measured on Day 14 as previously described (Zhang et al. 2005). Briefly, the saphenous vein was punctured using a 23‐gauge needle, and one drop of blood was collected using a 20‐*μ*L capillary glass (Drummond Scientific Inc.). One side of the tube was sealed with Hemato‐Seal and then centrifuged for 2 min in a Thermo IEC (Boston, MA) microcentrifuge. The lengths of red blood cells and plasma were measured.

### Enzyme immunoassay

Urinary sPRR was determined using a commercially sPRR enzyme immunoassay (EIA) kit (#JP27782, Immuno‐Biological Laboratories) according to the manufacturer's instructions.

### Histology

Under anesthesia, kidneys were harvested and fixed with 10% neutral buffered formalin. The tissues were subsequently embedded in paraffin, and 4‐*μ*m sections were cut and stained with periodic acid–schiff as previously described (Wang et al. 2015).

### Immunohistochemical staining

The tissues were fixed in 10% neutral buffered formalin for 24 h and then embedded in paraffin. After deparaffinization, thin sections (4 *μ*m) were processed for immunohistochemical staining. The slides were blocked with 1% bovine serum albumin for 1 h and were then incubated with the UCP1 antibody (1:100, Cat#: ab10983, Abcam, Cambridge, MA) at 4°C overnight. After washing off the primary antibody, sections were incubated for 1 h at room temperature with goat anti‐rabbit‐IgG‐ HRP (1:200, Cat#: sc‐2004, Santa Cruz Biotechnology, Santa Cruz, CA). The secondary antibody was washed off, followed by shaking off excess fluid. Freshly DAB Chromogenic substrate reagent (Cat#: AR1022, Boster Biological Technology, Pleasanton, CA) was added to cover the tissue section and incubated for 5 min. After rinsing with distilled water, the slide was placed in a bath of Mayer's hematoxylin for 1 min. The slide was rinsed under running tap water for 5 min and then mounted using glycerol gelatin. The images were taken with the microscope.

### Statistical analysis

Data are presented as mean ± SEM. Sample sizes were determined on the basis of previous or pilot experiments. Statistical analyses were performed using one‐way ANOVA with the Bonferroni test for comparing among three means or by paired or unpaired Student' *t*‐test for comparing between two means. Significance was accepted when *P *<* *0.05.

## Results

### sPRR‐His treatment does not attenuate indices of LiCl‐induced NDI

**Figure 1:**
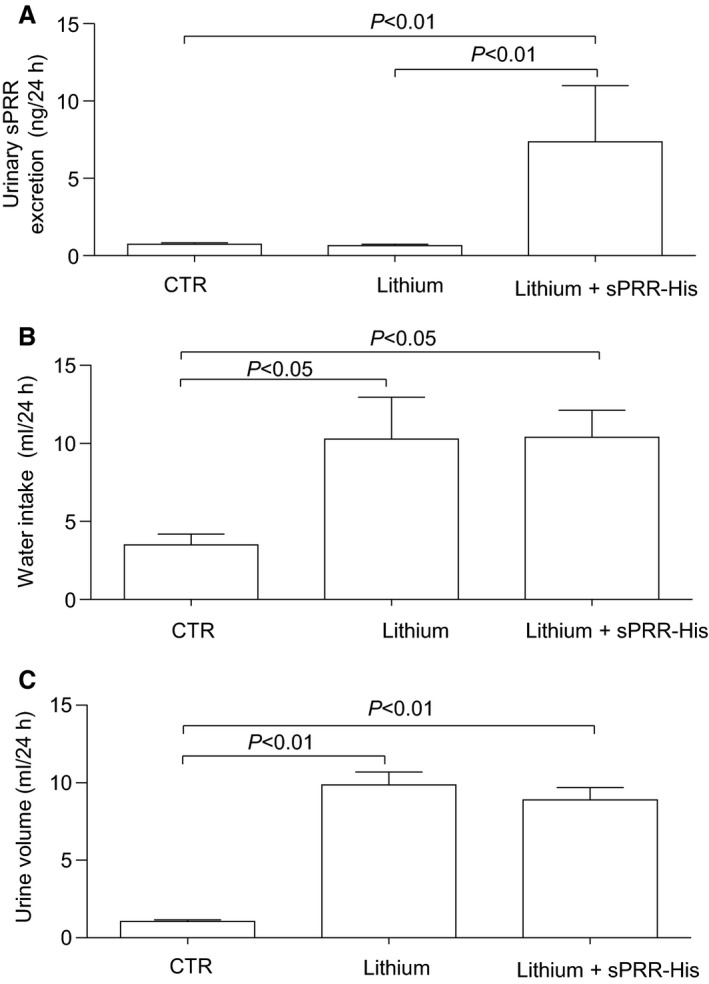
Effects of sPRR‐His on urinary sPRR excretion, water intake, and urine volume in lithium loaded mice. Male C57/BL6 mice were treated with vehicle, LiCl alone or in combination with sPRR‐His for 14 d. At the end of the experiment, animals were placed in metabolic cages for 24‐h urine collection. (A) ELISA deletion of urinary sPRR excretion (*n *=* *5 mice per group). (B) Water intake (*n *=* *10 mice per group). (C) Urine volume (*n *=* *10 mice per group). Data are mean ± SE.

**Figure 2:**
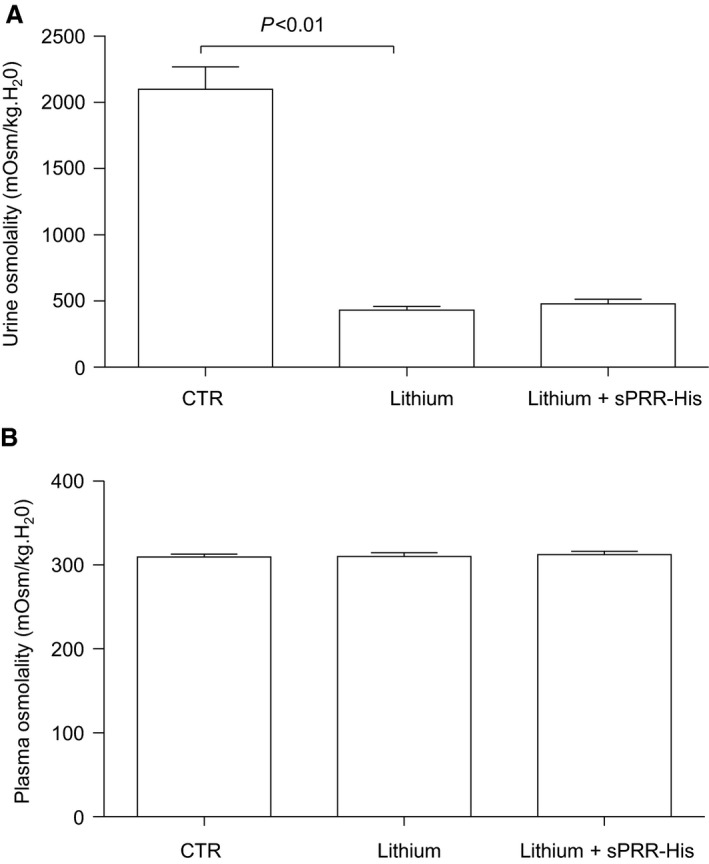
Effects of sPRR‐His on osmolalities in urine and plasma in lithium loaded mice. (A) Urine osmolality (*n *=* *5 mice per group). (B) Plasma osmolality (*n *=* *5 mice per group). Data are mean ± SE.

This study was designed to test the effect of sPRR‐His on lithium‐induced NDI. As expected, 24‐h urine collection on day 14 indicated that sPRR excretion was elevated in sPRR‐His treated mice versus the control and vehicle‐treated animals (Fig. 1A). However, urinary sPRR excretion remained unchanged in lithium loaded mice as compared with the controls (Fig. 1A). In support of previous studies that LiCl evokes indices of NDI, we observed increased urine volume (9.86 ± 0.82 vs. 1.06 ± 0.09, *P *<* *0.01) and water intake (12.2 ± 0.78 vs. 3.06 ± 0.14, *P *<* *0.01), and decreased urine (429.6 ± 29.3 vs. 2098.4 ± 170.2, *P *<* *0.01) but not plasma (310.0 ± 4.9 vs. 309.4 ± 3.5, *P *<* *0.01) osmolality, in LiCl treated vs. control mice, respectively. In contrast to our hypothesis, LiCl‐evoked NDI was refractory to concurrent administration of sPRR‐His (Fig. 2).

**Figure 3:**
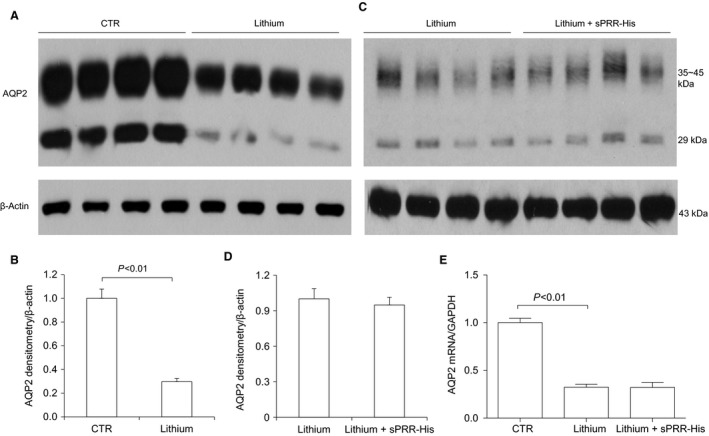
Effect of sPRR‐His on AQP2 expression in lithium loaded mice. Renal AQP2 expression was determined by immunoblotting analysis (A–D) and qRT‐PCR (E). (A) Representative blot for comparison between CTR and lithium groups. (B) Densitometric analysis of the data in (A) (*n* = 10 per group). (C) Representative blots for comparison between lithium and lithium + sPRR‐His groups. (D) Densitometric analysis of the data in (C) (*n* = 10 per group). (*E*) qRT‐PCR analysis of renal AQP2 mRNA expression (*n* = 5 per group). Data are mean ± SE.

AQP2 is a major water channel on the apical membrane of the CD (Nielsen et al. 2002). Downregulation of renal AQP2 contributes to lithium‐induced NDI, and our results from immunoblotting and qRT‐PCR analyses support this. Specifically, immunoblotting detected non‐glycosylated (29 kDa) and glycosylated (35–45 kDa) AQP2 in the kidney. As expected, the renal protein abundance of both AQP2 forms was suppressed in lithium loaded mice (Fig. 3 A and B). However, sPRR‐His treatment failed to affect lithium‐induced reduction in AQP2 protein (Fig. 3 C and D). Since both AQP2 forms showed a similar pattern, their densitometry data were combined (Fig. 3 B and D). Together, we observed no significant effect of sPRR‐His on lithium‐induced NDI.

### sPRR‐His treatment evokes beiging of WAT

**Figure 4:**
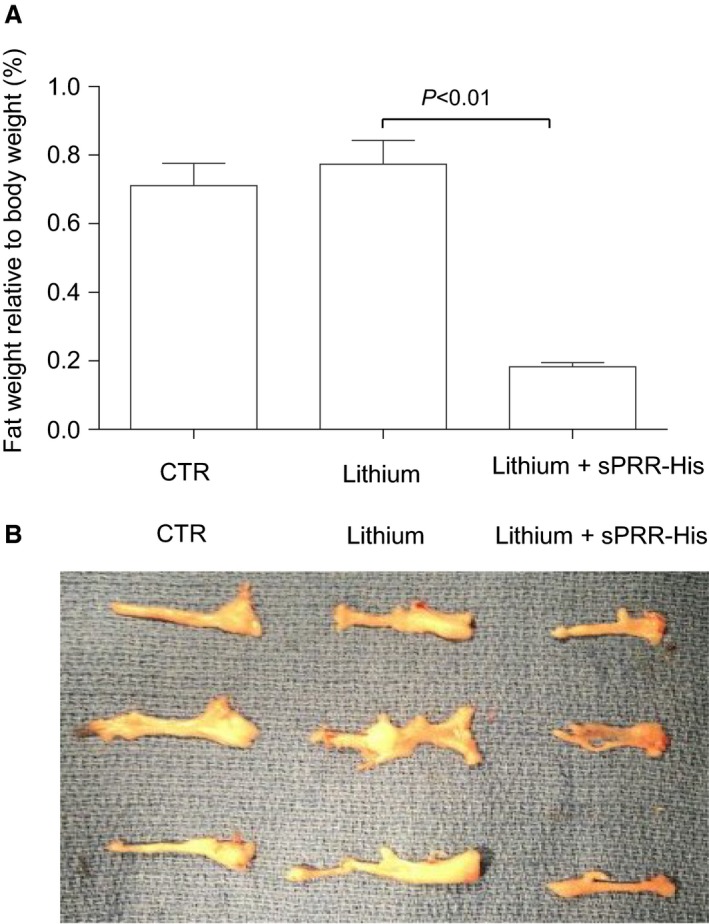
Effects of sPRR‐His on epididymal fat mass in lithium loaded mice. (A) Epididymal fat weight relative to body weight (*n* = 10 per group). Data are mean ± SE. (B) Images showing gross appearance of epididymal fat from three representative animals.

**Figure 5:**
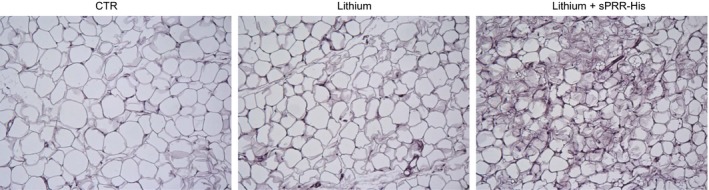
PAS staining of epididymal fat tissues. Shown is a representative image from five animals per group.

**Figure 6:**
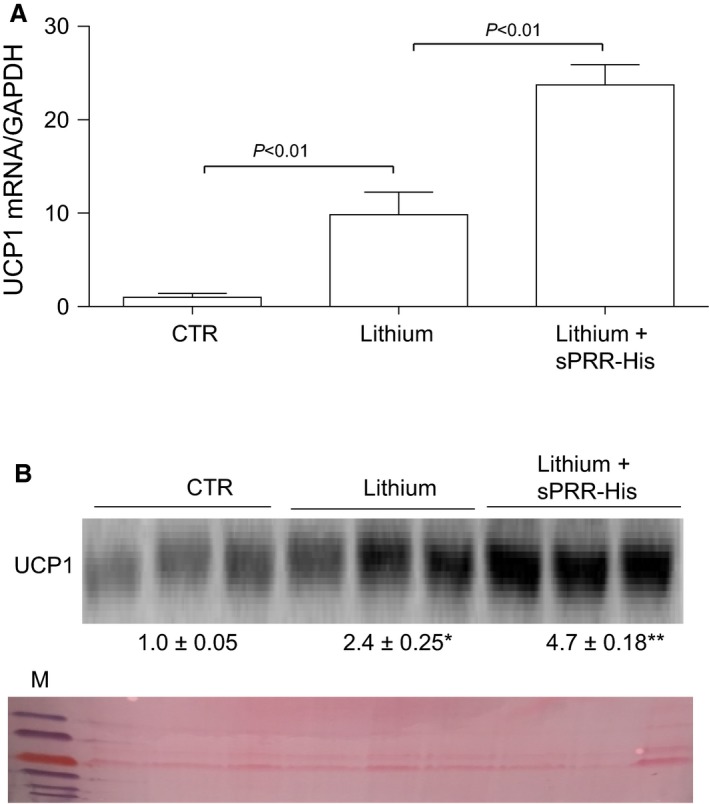
Effect of sPRR‐His on uncoupling protein 1 (UCP1) expression in epididymal fat. (A) UCP1 expression was determined by immunoblotting analysis and qRT‐PCR. (A) Effect of sPRR‐His on lithium‐induced UCP1 mRNA expression (*n* = 5 per group). (B) Effect of sPRR‐His on lithium‐induced UCP1 protein expression (*n* = 10 per group). *, *P *<* *0.01 versus CTR; **, *P *<* *0.01 versus Lithium alone. Ponceau staining serves as a loading control.

**Figure 7:**
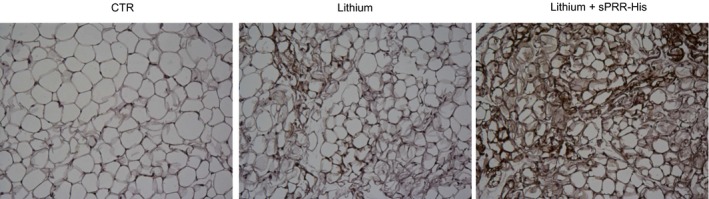
Immunohistochemistry of uncoupling protein 1 UCP1 in epididymal fat. The paraffin sections of the epididymal fat from CTR, Lithium, and Lithium + sPRR‐His groups were stained with anti‐UCP1 antibody. Shown is a representative image from five animals per group.

We were surprised to observe changes in WAT from mice with NDI treated with s sPRR‐His versus vehicle. For example, the epididymal fat mass was lower in LiCl‐fed mice treated with sPRR‐His versus vehicle. Of significant note, clusters of UCP1‐expressing adipocytes observed in mice that received LiCl+ vehicle were increased in animals wherein sPRR‐His was administered. Similar to adipocytes in BAT, beige cells in WAT are characterized by their multilocular lipid droplet morphology, high mitochondrial content and expression of markers of BAT such as UCP1. These adipocytes play a key role in regulation of thermogenesis in mammals, promoting energy expenditure and thus combatting metabolic disease (Chu and Gawronska‐Kozak 2017). Based on this unanticipated observation, (Fig. 4) we examined the extent of the beiging of WAT. PAS staining revealed remarkable differences in morphology of epididymal fat among the three groups (Fig. 5). By qRT‐PCR, UCP1 mRNA exhibited a 9.8‐fold increase in the lithium group and a 23.7‐fold increase in the lithium + sPRR‐His group, compared to the controls. This finding was subsequently confirmed at protein level. For example, relative to Con mice, UCP1 protein abundance was elevated 2.4‐fold or 4.7‐fold in lithium‐treated mice that were concurrently infused with vehicle or sPRR‐His, respectively. (Fig. 6). Finally, immunostaining was conducted to determine the protein level and localization of UCP1 protein. As compared to Con mice, the increase in UCP1 labeling observed in lithium‐fed mice infused with vehicle was exacerbated by infusion with sPRR‐His (Fig. 7).

## Discussion

The PRR undergoes protease‐mediated cleavage to produce a sPRR which can be detected in biological fluids by enzyme‐linked immunoassay. Circulating sPRR is elevated in patients with heart failure (Fukushima et al. 2013), kidney disease (Hamada et al. 2013),(Watanabe et al. 2013), hypertension (Morimoto et al. 2014), and preeclampsia, (Watanabe et al. 2012) and thus is widely considered to be a relevant biomarker of disease. The enzymatic source of sPRR appears complex involving furin, ADAM19, and recently site‐1 protease (Nakagawa et al. 2017). We reported earlier that the recombinant soluble (pro)renin receptor sPRR‐His upregulates renal aquoporin‐2 (AQP2) expression secondary to activation of frizzled‐8‐dependent *β*‐catenin signaling, and attenuates polyuria associated with NDI induced by V2R antagonism (Lu et al. 2016). Intercalated cells of the CD have a robust expression of PRR. As such, we propose that sPRR might be secreted from intercalated cells and act in a paracrine manner on neighboring principal cells to regulate the transport process. This new pathway has potential to play a major role in determining urine concentrating capability.

In this study we explored whether sPRR‐His might be beneficial concerning another clinically relevant context that is, the treatment of a lithium‐induced urine concentrating defect–a common form of acquired NDI. While lithium treatment precipitated the expected changes in water intake, urine volume, urine osmolality, and renal AQP2 expression, all responses were refractory to sPRR‐His administration. However, an unexpected finding of clinical importance was that sPRR‐His remarkably lowered fat mass via a conversion from WAT to BAT in mice with lithium‐induced NDI.

Central diabetes insipidus (CDI) results from damage to the pituitary gland that affects vasopressin storage and release. One treatment for CDI involves desmopressin, a selective V2R agonist, to replace the missing hormone. NDI is caused by failure of the kidney to respond to vasopressin due to a defective V2R, or signaling pathways distal to the V2R such as AQP2. NDI results from genetic abnormalities with 90% of the cases attributable to V2R or AQP2 mutations. With regard to this study, NDI also can develop in response to chronic lithium therapy.

Patients with extreme forms of NDI produce 20 L of urine per day, and experience severe dehydration that can result in death. Of the therapies available to treat NDI that is, a thiazide diuretic, a very low salt diet, and indomethacin, all are minimally effective. Therefore, novel therapeutic interventions are required. One strategy to improve a patients quality of life is to reduce the marked elevation in urine production. Accumulating evidence from our laboratory and others suggests that manipulating the prorenin/PRR pathway might be effective in this regard. For instance, administering sPRR‐His, a recombinant sPRR, effectively attenuated urine output in a murine model of NDI that was induced by V2R antagonism (Lu et al. 2016). These findings support a physiological role of the sPRR in regulating water homeostasis, and provide solid evidence that manipulating this target has therapeutic value in the treatment of NDI secondary to a V2R defect.

In contrast to the originally stated hypothesis, none of the abnormalities that are characteristic of our murine model of lithium‐induced NDI that is, polydipsia, polyuria, hypoosmotic urine, and reduced renal AQP2 mRNA and protein expression, were beneficially altered by sPRR‐His treatment. The lack of an antidiuretic effect of sPRR‐His in NDI evoked by lithium‐loading versus V2R antagonism is unlikely due to insufficient dosage or inappropriate mode of delivery (Lu et al. 2016), but does suggest distinct roles of the sPRR in the two different contexts of NDI that we have evaluated. In this regard, NDI induced by V2R inhibition is less complex relative to NDI induced by lithium loading. For example, lithium inhibits glycogen synthase 3*β*, resulting in the release of prostaglandins that reduce adenylyl cyclase activity, cyclic AMP production, and AQP2 phosphorylation (Rao et al. 2005). Second, lithium activates purinergic signaling which is well known to induce diuresis and natriuresis (Rao et al. 2005). Third, a long‐term lithium treatment induces renal interstitial fibrosis, which may indirectly affect urine concentrating capability (Markowitz et al. 2000; Nielsen et al. 2008a). Indeed, results from microarray and proteomic screening analyses indicate that lithium treatment alters renal medullary expression of a large number of transcripts and proteins, particularly those involved in cellular proliferation and cytoskeletal organization (Christensen et al. 2006, 2004; Kim et al. 2003; Nielsen et al. 2008a; Rojek et al. 2005). Despite a lack of data to explain the divergent results, it is clear that sPRR‐His is an efficacious treatment for NDI that is precipitated by V2R antagonism but not lithium administration.

Our finding that sPRR‐His treatment reduced epididymal fat mass and precipitated a beiging of this depot in mice with lithium‐induced NDI was unexpected, and represents a novel and previously unappreciated role for the sPRR in metabolism. Of note, adipocyte‐specific deletion of sPRR in mice results in lipodystrophy, and prevents diet‐induced obesity that is accompanied by a paradoxical increase in plasma sPRR (Wu et al. 2016). While the mechanism for this response is unknown, it is possible that elevated plasma sPRR in adipocyte specific null mice may contribute to the development of lipodystrophy in these animals. Similarly unknown is the signaling pathway that confers anti‐adipogenenic action of sPRR. The downstream signaling pathways coupling PRR contain *β*‐catenin, MAPK, and Nox‐4‐derived H_2_O_2_ (Lu et al. 2015; Oshima et al. 2014; Yang 2017). Among these signaling pathways, *β*‐catenin signaling is reported to be a negative regulator of adipogenesis ((Dogan et al. 2017; Jeon et al. 2016; Lee et al. 2017). We demonstrated sPRR stimulation of *β*‐catenin signaling in our previous study (Lu et al. 2016). Therefore, it seems possible that the anti‐adipogenic action of sPRR may be attributed to the increased *β*‐catenin activity.

High‐dose lithium treatment to chicks decreased abdominal adipose tissue after 35 but not 14 days. (Bai et al. 2017) Our results are congruent with these findings that is, a 14 day regimen of lithium feeding did not alter fat mass. However, a significant reduction in fat mass, together with increased multilocular lipid droplet morphology, high mitochondrial content, and mRNA and protein expression of UCP1 in adipocytes, was observed in mice that consumed lithium for 14 days concurrent with sPRR‐His administration for the final 7 days. These findings suggest that a beneficial relationship between lithium and sPRR‐His exists concerning adipocyte metabolism in the context of NDI. The nature of this relationship is unknown but may be related to a common downstream mediator of *β*‐catenin signaling albeit through different mechanisms. sPRR activates *β*‐catenin signaling via frizzle‐8 (Lu et al. 2016) whereas lithium inhibits glycogen synthase kinase 3 (GSK‐3), resulting in nuclear translocation of unphosphorylated *β*‐catenin (Jope 2003). The different upstream mechanisms may be the basis for the synergistic effects of the two agents on *β*‐catenin activation and thus energy metabolism.

In summary, this study tested the hypothesis that sPRR‐His treatment to mice with lithium‐induced NDI would alleviate the hallmark characteristics of this disease, but the data were not supportive. Instead, an unanticipated finding was that sPRR‐His treatment in lithium loaded mice markedly reduced epididymal fat mass and induced a phenotypic switch from WAT to BAT in this depot. These data provide the first evidence that manipulating the sPRR in the context of lithium‐induced NDI has a novel metabolic benefit.

## Conflict of Interest

None declared.
